# Retraction: Gemcitabine aggravates miR-199a-5p-mediated breast cancer cell apoptosis by promoting VEGFA downregulation *via* inactivating the AKT signaling pathway

**DOI:** 10.1039/d1ra90114a

**Published:** 2021-05-05

**Authors:** Laura Fisher

**Affiliations:** Royal Society of Chemistry Thomas Graham House, Science Park, Milton Road Cambridge CB4 0WF UK advances-rsc@rsc.org

## Abstract

Retraction of ‘Gemcitabine aggravates miR-199a-5p-mediated breast cancer cell apoptosis by promoting VEGFA downregulation *via* inactivating the AKT signaling pathway’ by Dingmei Deng *et al.*, *RSC Adv.*, 2019, **9**, 20385–20394, DOI: 10.1039/C9RA00016J.

The Royal Society of Chemistry hereby wholly retracts this *RSC Advances* article due to concerns with the reliability of the data. The images in the article, and the raw data provided by the authors, were screened by an image integrity expert.

All the western blots in the article contain very unusually regular-shaped bands. The raw data provided for the western blot images does not appear to be genuine. There are traces of cloning in the background of the raw data for multiple panels, including Fig. 3 (Bax-MCF-7 and Cle-caspase-3-MCF-7) and Fig. 4 (VEGFA-MCF-7), indicating that the raw data was manipulated. In addition, for a number of panels, the area immediately around the bands in the raw data does not exactly match the published images. The bands in the figure panels also appear closer to each other than in the raw data. The raw data provided by the authors, therefore, cannot be used to validate the published data.

Given the significance of the concerns about the validity of both the data in the article and the raw data provided by the authors, the findings in this paper are not reliable.

The authors agree to the retraction.

Signed: Laura Fisher, Executive Editor, *RSC Advances*

Date: 9^th^ April 2021

## Supplementary Material

